# Clinical Research Support Activities in Core Clinical Research Hospitals Throughout Japan

**DOI:** 10.14789/jmj.JMJ22-0011-P

**Published:** 2022-07-14

**Authors:** YUJI NISHIZAKI, RIEKO UEDA, SHUKO NOJIRI, SHOJI SANADA

**Affiliations:** 1 Division of Medical Education, Juntendo University School of Medicine, Tokyo, Japan; 1 Division of Medical Education, Juntendo University School of Medicine, Tokyo, Japan; 2 Medical Technology Innovation Center, Juntendo University, Tokyo, Japan; 2 Medical Technology Innovation Center, Juntendo University, Tokyo, Japan; 3 Clinical and Translational Research Center, Kobe University Hospital, Kobe, Japan; 3 Clinical and Translational Research Center, Kobe University Hospital, Kobe, Japan

**Keywords:** clinical research support, core clinical research hospital, the Japan Registry of Clinical Trials (jRCT)

## Abstract

We evaluated the current status of supporting activities in core clinical research hospitals across Japan using the Japan Registry of Clinical Trials (jRCT) data given the lack of previous research objectively investigated supporting activities for clinical trials in core clinical research hospitals throughout Japan. Briefly, our findings showed that despite the officially supposed cooperation scheme, core clinical research hospitals have not been the primarily selected contractor for clinical research supporters. Considering that core clinical research hospitals are designated spearhead physician-led clinical trials and are responsible for ensuring the quality of such trials, there is a need to determine why the officially supposed cooperative scheme between core and non-core hospitals are still not established in Japan in order to increase the development and quality of Japanese clinical research with maximum efficiency moving forward.

## Core clinical research hospital

In Japan, the Medical Care Act has established core clinical research hospitals that are designated to play a leading role in physician-led clinical trials. Moreover, they are responsible for the development of clinical research aimed at uncovering novel innovative pharmaceuticals and medical instruments in Japan according to international standards. These hospitals are also mandated to support clinical research developed in other medical institutions^1-2)^. The Clinical Research Act, which came into effect on April 1, 2018 in Japan^3)^, seeks to prevent research misconduct and ensure the reliability of the clinical studies for not only the research subjects but also the general public. As stipulated by the mentioned legislation, the implementation plans and research outlines of clinical trials are required to be registered with the Japan Registry of Clinical Trials (jRCT) database prepared by the Ministry of Health, Labor and Welfare^4)^. The clinical trial implementation system includes information on the principal investigator, co-investigators, program manager, study manager, data managers, monitors, biostatisticians, and auditors^5)^.

## Clinical research support activities in core clinical research hospitals

To the best of our knowledge, no previous research has objectively investigated supporting activities for clinical trials in core clinical research hospitals throughout Japan. Therefore, we sought to evaluate the current status of supporting activities in core clinical research hospitals across Japan using jRCT data. Data for a total of 860 hospitals registered in the jRCT from April 1, 2018 to May 31, 2019, including non-core clinical research hospitals, were reviewed, excluding unknown data. We investigated the type of institutions from which non-clinical research core hospitals would request clinical research support in conducting clinical trials.

**Figure 1:**
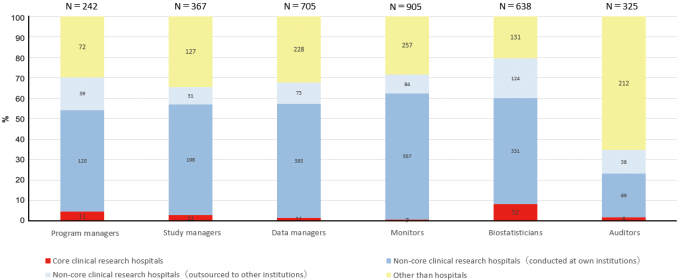
The rate of contact requests by non-core clinical research hospitals and information of outsourcers for clinical research supporters.

The results of our survey are summarized in Figure 1. The rate of contact requests by non-core clinical research hospitals to core clinical hospitals for each clinical research supporter is as follows: program manager (1.3%, 11/860), study manager (1.3%, 11/860), data managers (1.2%, 11/903), monitors (0.8%, 7/913), biostatisticians (6.0%, 52/860), and auditors (0.7%, 6/865). It has become clear that core clinical research hospitals have not been primarily selected as a contractor for all clinical research supporters, despite the officially supposed cooperation scheme. Aside from auditors, non-clinical research core hospitals requested support from the clinical research support department of their own institutions.

## Conclusions

The core clinical research hospital has primarily been designated to lead clinical trials in Japan. It is important for core clinical research hospitals to conduct secure and high-quality clinical research by themselves while also supporting clinical research conducted by neighboring non-core hospitals. We believe that by channeling clinical research conducted throughout Japan through core clinical research hospitals, the quality of clinical research in Japan will improve considerably. Additionally, patients who wish to participate in clinical trials will be guided by the existence of landmarks, namely core clinical research hospitals nationwide. Furthermore, core research hospitals with several on-going high-quality clinical research studies might effectively train and develop numerous young physicians and researchers who want to enrich their experience on clinical research in the near future. As such, there is a need to determine why the officially supposed cooperative scheme between core and non-core hospitals are still not established in Japan in order to increase the development and quality of Japanese clinical research with maximum efficiency moving forward.

We speculate that one of the major causes of the lack of an established cooperation between core and non-core clinical research hospitals is a mismatch between needs and feeds. The core clinical research hospitals have built a system to provide segmented research support, including program managers, study managers, data managers, monitors, biostatisticians, auditors, and clinical research coordinators. However, we infer that researchers from non-core clinical research hospitals would need multifunctional comprehensive support rather than segmented support.

## Funding

This study was supported by a grant from the Japan Agency for Medical Research and Development (AMED) under Grant Number 19lk1903002 h0002.

## Author contributions

YN and RU have designed this study as a whole and written this manuscript. SN has contributed to statistical analyses. YN, RU, and SS have contributed to data collection. All authors have contributed, provide advice on the interpretation of the results, and approved the final manuscript.

## Conflicts of interest statement

There are no conflicts of interest to declare.

## References

[1] Ministry of Health, Labour and Welfare. About publication of business report of core clinical research hospitals [Internet] [cited 7 Aug 2021]. Available from: https://www.mhlw.go.jp/stf/seisakunitsuite/bunya/0000165585.html. (in Japanese).

[2] Ueda R, Nishizaki Y, Homma Y, et al: Importance of quality assessment in clinical research in Japan. Front Pharmacol, 2019; 10: 1228. 31680985 10.3389/fphar.2019.01228 PMC6814083

[3] Ministry of Health, Labour and Welfare. About publication of the Clinical Research Act [Internet] [cited 7 Aug 2021]. Available from: https://www.mhlw.go.jp/stf/seisakunitsuite/bunya/0000163417.html. (in Japanese).

[4] Ministry of Health, Labour and Welfare. About publication of jRCT (Japan Registry of Clinical Trials) [Internet] [cited 7 Aug 2021]. Available from: https://jrct.niph.go.jp (in Japanese).

[5] Ueda K, Uemura N, Matsuyama K, et al: Performance index for types of clinical research support service providers for academic research organizations in Japan: a cross-sectional survey. Clin Transl Sci, 2021; 14: 745-55. 33278325 10.1111/cts.12942 PMC7993270

